# Supplementary material for: Identification of sSIGLEC5 and sLAG3 as New Relapse Predictors in Lung Cancer
Source: Biomedicines. 2022 Apr 30;10(5):1047. doi: 10.3390/biomedicines10051047 (PMC9139133; doi:10.3390/biomedicines10051047)
Supplement: Supplementary file 1 [file biomedicines-10-01047-s001.zip › Supplementary Table S1.pdf]

Supplementary Table S1. Logistic regression model for relapse prediction in Lung Cancer.

Variables in the equation	B	SD	Wald	P-value	OR	OR CI 95	
						Low	High
**sLAG3 (pg/mL)**	0.001606	0.000516	9.656418	0.001886	1.001607	1.000593	1.002622
**sSiglec5 (ng/mL)**	0.003875	0.002010	3.717969	0.053829	1.003883	0.999936	1.007846

Wald intro logistic regression, including sLAG3 and sSiglec5. Units: sLAG3 in pg/mL and sSiglec5 in ng/mL.

B, B weight coefficient; SD, standard deviation of B; Wald, Wald statistic; OR, odds ratio; OR CI 95, 95% confidence interval of odds ratio.
