# Supplementary figures and images for: Identification of sSIGLEC5 and sLAG3 as New Relapse Predictors in Lung Cancer
Source: Biomedicines. 2022 Apr 30;10(5):1047. doi: 10.3390/biomedicines10051047 (PMC9139133; doi:10.3390/biomedicines10051047)

Supplementary Figure S1

(A)

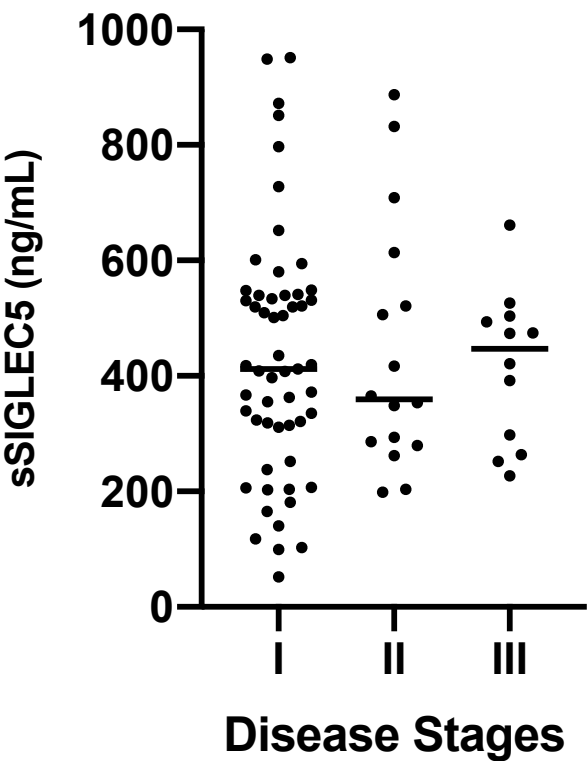

(B)

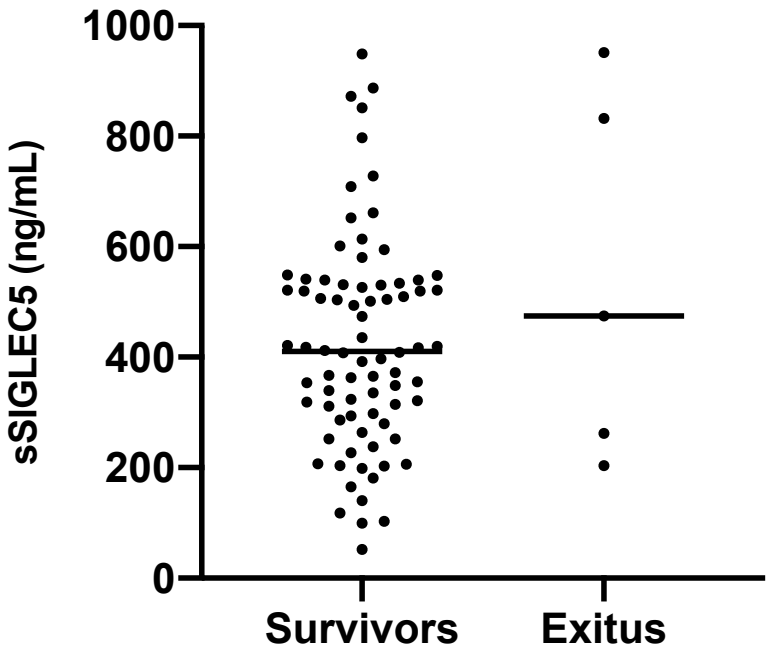

Supplement: Supplementary file 1 [file biomedicines-10-01047-s001.zip › Supplementary Figure S1.pdf]
